# Inter-annual variation in prevalence and intensity of mite parasitism relates to appearance and expression of damselfly resistance

**DOI:** 10.1186/1472-6785-10-5

**Published:** 2010-02-14

**Authors:** Laura Nagel, Tonia Robb, Mark R Forbes

**Affiliations:** 1 Department of Biology, Carleton University, Ottawa, Canada

## Abstract

**Background:**

Insects can resist parasites using the costly process of melanotic encapsulation. This form of physiological resistance has been studied under laboratory conditions, but the abiotic and biotic factors affecting resistance in natural insect populations are not well understood. Mite parasitism of damselflies was studied in a temperate damselfly population over seven seasons to determine if melanotic encapsulation of mite feeding tubes was related to degree of parasitism, host sex, host size, emergence timing, duration of the emergence period, and average daily air temperature.

**Results:**

Although parasite prevalence in newly emerged damselflies was > 77% each year, hosts did not resist mites in the early years of study. Resistance began the year that there was a dramatic increase in the number of mites on newly emerged damselflies. Resistance continued to be correlated with mite prevalence and intensity throughout the seven-year study. However, the percentage of hosts resisting only ranged from 0-13% among years and resistance was not sex-biased and was not correlated with host size. Resistance also was not correlated with air temperature or with timing or duration of damselfly emergence.

**Conclusions:**

Resistance in host damselflies was weakly and variably expressed over the study period. Factors such as temperature, which have been identified in laboratory studies as contributing to resistance by similar hosts, can be irrelevant in natural populations. This lack of temperature effect may be due to the narrow range in temperatures observed at host emergence among years. Degree of mite parasitism predicted both the appearance and continued expression of resistance among parasitized damselflies.

## Background

The factors that cause variation in parasitism and host resistance in natural populations have important implications for the ecology and evolution of both hosts and parasites. Characteristics such as host population size have long been known to affect parasite population dynamics [1], and attention has recently turned to the effects of host sex and age on parasitism and resistance (reviewed by [2]). The effects of abiotic factors such as temperature and precipitation on host-parasite interactions also are increasingly being recognized [3-6] and might have effects on whether or not resistance to parasites is expressed.

Host resistance to parasites can be behavioral, physiological or mechanical. Not all host species or populations respond immunologically to parasitism [7,8], and there can be variation in response within a species (e.g., between sexes [9]). Physiological resistance by invertebrates is commonly a cell-mediated response that involves encapsulation around a foreign invader by haemocytes. The pro-phenoloxidase enzymatic cascade then kills the parasite through the synthesis of melanin, which has cytotoxic and antimicrobial properties [10,11]. This type of resistance has been shown to vary due to biotic [10,11] and abiotic [12-15] factors, and can have a heritable component [12,16,17]. Our knowledge of the genetic basis of resistance in animals is increasing [18-21], but the degree to which resistance can be a plastic response to environmental variation in natural populations is not well understood.

Maintaining immune defenses is costly [22,23], and therefore subject to evolutionary tradeoffs with other important traits, such as competitive ability, reproductive success and survival. These tradeoffs have been demonstrated in many laboratory studies [24-35] that have added to our understanding of life history evolution and immune defense. However, the factors affecting resistance may differ under natural conditions; resistance variation might depend on temperature, resource availability and host and parasite abundance [2,35].

In temperate damselflies, seasonal variation in body size and timing of emergence can be associated with environmental factors such as air temperature and photoperiod [36]. There is also seasonal and inter-annual variation in parasitism by mites [37]. Larval water mites can be resisted physiologically by their odonate hosts, shortly after hosts eclose from their aquatic larval stage. If melanotic encapsulation of the mite feeding tube occurs, it is initiated within 24 h of host emergence. There is evidence that melanotic encapsulation is affected by air temperature under controlled conditions [14,15]. It is also known that the effects of engorging mites on odonates can be severe, as they can affect perching and flight [38,39], cause cellular and tissue damage [40], reduce body fat content [41], reduce mating success [42] and lower fecundity [38,42].

We examined host resistance to a specialist mite in a damselfly population over seven seasons to determine whether variation in abiotic and biotic factors was correlated with variation in resistance. Larvae of the mite *Arrenurus pollictus *are phoretic on the final aquatic larval instars of the damselfly *Lestes disjunctus*. When the damselflies eclose, the mites pierce the host cuticle and begin to feed. When damselflies return to water to mate and oviposit, the fully engorged mites drop off their hosts to complete their development in the water.

We measured mite prevalence (the percentage of individuals in the sample that were parasitized by one or more mites) and intensity (the number of parasites per infested host, following [43]) on hosts from 2002-2008. We monitored damselfly emergence timing and wing length (a correlate of body size). We also recorded air temperature before emergence and during the flight period each year.

## Results

### Emergence of pre-reproductive damselflies

**Figure 1:**
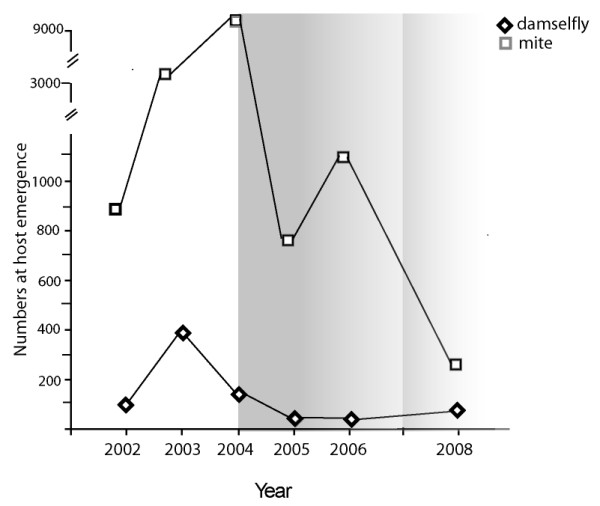
**Numbers of *Lestes disjunctus *damselflies and *Arrenurus pollictus *mites trapped during damselfly emergence over a 7-year period**. Darkest shading indicates years when resistance levels in the mature damselfly population were highest (> 9.5%; see Figure 2). No shading indicates years with no resistance. Emergence traps were not used in 2007.

The numbers of newly emerged damselflies caught in emergence traps varied considerably among years (analyses were based on 6 years of data, as emergence traps were not deployed in 2007). 2003 was an anomalous year with more than twice the average number of the other years (Additional file 1: Table S1; Figure 1). It was also the only year with a female biased sex ratio (*χ*^2 ^= 11.78, *p *= 0.0006). There were no differences between the sexes in the starting dates or durations of the emergence period, so single yearly values are reported in Additional file 1: Table S1.

Numbers of parasites on hosts caught in emergence traps varied considerably over the study period (Figure 1). The total numbers of mites ranged from a low of 310 (in 2008 for 75 hosts) to a high of 9174 (for 169 hosts in 2004). A dramatic increase in the parasite population size from 2002-2004 was followed by a decrease in host numbers in 2004 and 2005, which was followed by a decline in parasite numbers in 2005. Resistance first appeared in the host population in 2004, as parasite numbers reached their peak (and host numbers were declining; Figure 1).

**Figure 2:**
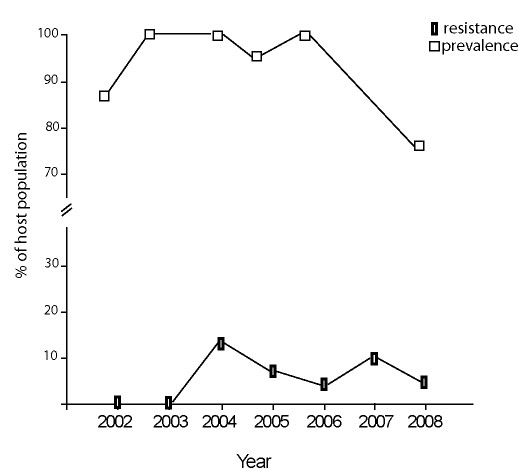
**Prevalence (percentage of the sample of host population infested) of mites in newly emerged *Lestes disjunctus *damselflies and the percentage of the host population of mature damselflies that resisted mites over 7 years**. Prevalence was not measured in 2007.

Prevalence of mites in the *L. disjunctus *population increased from 86% to 100% in the second year of the study, and declined to 77% in the last year (Additional file 1: Table S1; Figure 2). This inter-annual variation in prevalence was significant (Log-linear *χ*^2 ^= 49.5, df = 5, *p *= 0.006). Although there was a trend for higher parasite prevalence in females than in males each year, this was not statistically significant except in 2008 (*χ*^2 ^= 5.21, *p *= 0.003). Females were larger than males each year based on wing lengths (used as a proxy of size (Additional file 1: Table S1)), and there was a significant, unexpected, increase in wing length in both females (*F*_1,469 _= 44.41, *p *< 0.0001) and males (*F*_1,338 _= 30.79, *p *< 0.0001; Additional file 1: Table S1) over the seven-year study period.

Intensity of parasitism varied yearly, and was highest in 2004 with an average of 54 mites per infested host (Additional file 1: Table S1). There was no significant sex bias in intensity in the first or the last year of the study, but infested females had significantly more mites than males in the other 4 years (which were also the years with prevalence near 100%; Average Difference= 9.07, *p *< 0.001 based on 1000 permutations in comparison of means; Mean Intensity: Males - 42.14 ± 0.13, N = 104, Females - 47.21 ± 1.61, N = 105). There was a significant effect of year and of sex in explaining variation in intensity over the study period (Year *F*_5,563 _= 4.69, *p *= 0.003; Sex *F*_1,563 _= 2.1, *p *= 0.04; interaction NS, based on 1000 permutations). Prevalence and intensity were not correlated with either air temperature prior to emergence or with emergence period timing.

### Mature damselflies

On average, 200 mature damselflies were caught each year in net surveys (Additional file 1: Table S2). Males usually outnumbered females, probably because of behavioural differences that made them more detectable. As was the case with the emergence trap samples, females were larger than males each year, and wing length increased over the study period in both males (*F*_1,610 _= 12.93, *p *= 0.0003) and females (*F*_1,530 _= 6.60, *p *= 0.01; data not shown).

Inter-annual variation in parasitism followed the same patterns as in newly emerged damselflies, with both prevalence (Log-linear *χ*^2 ^= 10.86, df = 5, *p *= 0.01) and intensity (*F*_1,510 _= 574.63, p < 0.0001) differing significantly among years. Unlike the pattern in newly emerged damselflies, there were no significant sex differences in intensity among years (Year *F*_5,326 _= 5.32, *p *= 0.005, Sex *F*_5,326 _= 0.50, *p *= 0.50, based on 1000 permutations). Prevalence in males was higher than in females in 3 years, but this was statistically significant only in 2002 (*χ*^2 ^= 6.37, *p *= 0.001).

### Resistance

**Figure 3:**
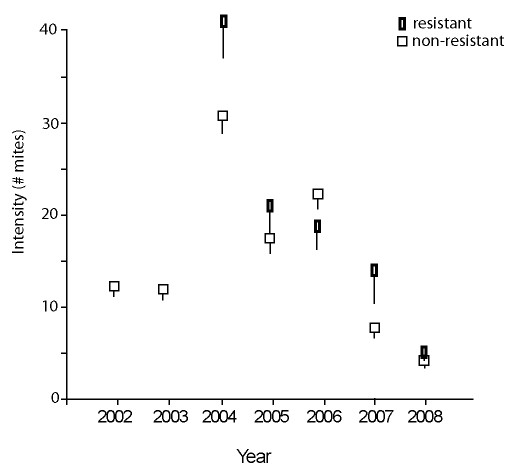
**Mean parasite intensity (number of *Arrenurus pollictus *mites per infested host (± s.e.)) on mature *Lestes disjunctus *damselflies that resisted or did not resist mites over 7 years**. Resistance was not evident in 2002 and 2003. See Additional file 1: Table S3 for sample sizes.

In 2002 and 2003, *L. disjunctus *did not mount a melanotic encapsulation resistance response to mites. No dead mites were seen on any of over 600 mature damselflies surveyed, despite the observers' extensive experience with field surveys of resistance in lestid damselflies (MRF was present each year, and TR and LN were the only other observers conducting surveys). In 2004, dead mites were found on hosts for the first time, and all damselflies with dead mites in the first two years after the appearance of resistance were killed and examined with a microscope. Each dead mite was associated with a feeding tube that had undergone melanotic encapsulation similar to that seen by the authors in a related damselfly, *Lestes forcipatus *[15,44,45]. The percentage of the damselfly population with dead mites was highest in 2004 (13% of the host population; shown as the lower line in Figure 2), the year when the highest intensities at emergence (Figure 3) and in mature damselflies were also seen. Resistance declined to 4% in the last year of the study (when intensity was lowest).

Damselflies that had resisted mites (i.e. that had one or more dead mites on them when captured) often also had live engorging mites. Although the most common pattern was to have 1 dead mite (and no live mites or scars, see methods), damselflies that resisted had a range from 0 live mites and 13 dead mites to 66 live mites and 1 dead mite. Although hosts with dead mites also tended to have high intensities (Additional file 1: Table S3; Figure 3), the probability of a damselfly having one or more dead mites was not significantly related to intensity within a year (2004 *χ*^2 ^= 65.4, *p *= 0.12; 2005 *χ*^2 ^= 49.3, *p *= 0.17; 2006 *χ*^2 ^= 62.7, *p *= 0.06; 2007 *χ*^2 ^= 20.4, *p *= 0.2; 2008 *χ*^2 ^= 12.9, *p *= 0.61). There were also no significant differences in sex or in wing length between those damselflies that resisted and those that did not within a year (Additional file 1: Table S3).

There was inter-annual variation in air temperature prior to the emergence period (*F*_1,813 _= 224.78, *p *< 0.0001). Inter-annual variation in air temperature was also apparent during the flight period (*F*_1,1350 _= 798.96, *p *< 0.0001), with 2005 being the warmest year (as was the case at damselfly emergence). The duration of the emergence period also differed significantly among years (*F*_1,813 _= 22.56, *p *< 0.0001; Additional file 1: Table S1). However, resistance was not affected by any of these factors.

**Figure 4:**
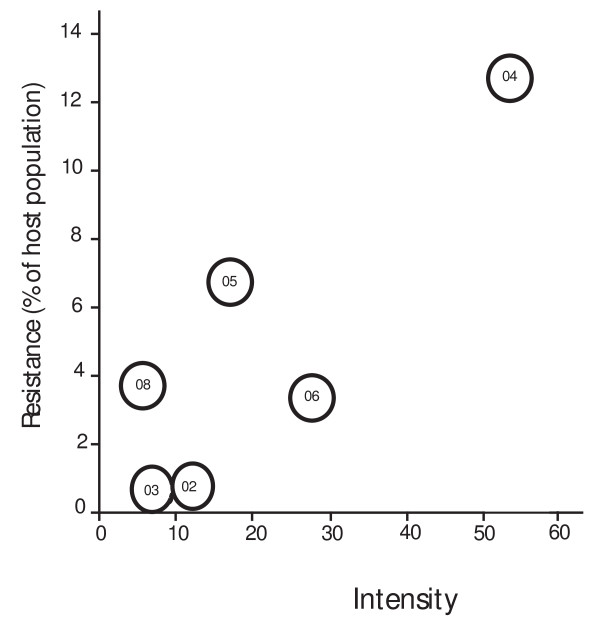
**Mean parasite intensity (number of *Arrenurus pollictus *mites per infested host) on *Lestes disjunctus *damselflies at emergence each year plotted against resistance (the % of the mature damselfly population that resisted mites) for 6 years of the 7-year study showing a significant relationship between these two measures**. Intensities (± s.e.) for each year are, 2002: 10.2 ± 0.9, 2003: 9.1 ± 0.4, 2004: 54.3 ± 1.8, 2005: 17.6 ± 2.4, 2006: 26.1 ± 2.8, 2008: 5.3 ± 0.5. Intensity was not measured in 2007.

Within each sex, wing length and mite intensity in the mature population were not significant predictors of resistance. However, when the sexes were grouped using data from 2004-2008, intensity was significantly correlated with the probability of mature damselflies resisting mites (intensity: *G *= 612.1, *p *< 0.001; year: *G *= 692, *p *< 0.001, interaction: *G *= 141.9, *p *< 0.001). Intensity in the newly emerged damselfly population sample was also significantly correlated with whether resistance occurred in the mature damselfly population over the study period (*G *= 161.7, df = 1, *p *< 0.0001; Figure 4).

## Discussion

Whether an individual damselfly resisted mites did not appear to be affected by its mite burden. Instead, investment in resistance seemed to be determined before parasitism occurred, and was largely based on the parasite population size. Mite intensity in newly emerged damselflies was highest in the year that resistance appeared in the host population, when 13% of mature damselflies surveyed had dead mites on them. This declined to 4% by the last year of the study, when mite prevalence and intensity were lowest. Observing this system over seven years allowed us to detect what appears to be a cycle of host resistance that is related to a cycle of parasite abundance.

The probability of hosts encountering parasites is generally thought to increase as host population size increases [46]. Natural selection should therefore favour a strategy in hosts of increasing investment in resistance when host numbers are high. Evidence for this comes from insects shown to invest more in immune defense when reared under parasite-free but crowded conditions compared to conspecifics reared at low host densities [46,47]. In the present study, resistance appeared in the host population the year after the highest numbers of damselflies were present in the emergence traps, which appears to support this scenario. However, it is unclear how high host numbers would lead to an increased probability of encountering parasites in this system since both host and parasite have one generation per season, and the parasitic phase of mites is limited to a small portion of their life cycle.

There is, however, a significant relationship (which can be seen in Figure 4) between mite intensity in newly emerged hosts and resistance in mature hosts across years. Furthermore, resistance appeared in the damselfly population the year that intensity at damselfly emergence was five times as high as the previous two years. This suggests that larval damselflies that encounter high densities of mites in the water just before emerging invest more in immune response immediately after emergence. Resistance in several other lestid damselfly species was also positively correlated with high prevalence and intensity of the mite *Arrenurus planus*, although that study took place over only one season [7]. A similar pattern was identified in a study showing that immune responses decreased with decreasing parasite abundance across bird populations [48].

The numbers of hosts and parasites captured during host emergence suggest that there is parasite-induced host mortality from year to year. For example, host numbers decreased in 2004 and 2005 after a dramatic increase in the parasite population size from 2002-2004. There also appears to be an effect of host numbers on the parasite population, as the decline in mite numbers from 2004 to 2005 coincides with a decline in available hosts, as well as with the appearance of resistance in the host population in 2004.

Our data suggest that resistance in these damselflies is not only affected by the parasite population in the current year, but that there may be a year delay before parasite prevalence affects resistance levels. For example, an increase in prevalence in 2003 is followed the next year by an increase in resistance in the host population; a decline in prevalence in 2005 is followed in 2006 by a decline in resistance (as seen in Figure 2). The mechanism for such an apparently adaptive response to inter-annual variation in parasitism is not known, but could have to do with maternal effects. Mitchell and Read [49] showed that maternal effects in *Daphnia magna *that were induced by environmental change were responsible for changes in resistance to bacteria in their offspring.

Resistance was not correlated with air temperature at emergence or with emergence period timing, although both of these abiotic factors were correlated with seasonal increases in resistance to the generalist mite *A. planus *in a related damselfly [15,44,50]. Temperature has also been identified as a factor affecting resistance in lab studies of fruit flies [24,51] and mosquitoes [52], both of which showed increased resistance when reared at higher temperatures. It is important to note that the temperature range used in these laboratory experiments was much greater than that found naturally occurring in this study (average daily temperature ranged only by 2.5 degrees C over the course of this study). In addition, a complex suite of factors involving temperature may affect patterns of parasitism and resistance in this system. For example, prevalence and intensity were high in years with early or short damselfly emergence periods, and air temperatures were high in these years. In addition, costs of parasitism may be greater at higher temperatures because mites may engorge more quickly at higher temperatures [15]. The temperature that damselflies are exposed to after mounting an immune response has also been shown to affect correlates of fitness [30]. Finally, temperature will affect resource availability, which has been shown to affect immune response in many insects [6,53].

Females tended to have higher mite prevalence and intensity than males, but there were no significant differences in resistance between sexes. In mite-odonate systems, there are several examples of sex biases in parasitism [54-58]. Some authors have suggested that males should invest less in resistance to parasites than females because males increase their reproductive success through mating success, not longevity (as females tend to) [2]. This view has been questioned by [59] (see [60] for a review). Several empirical studies with insects have failed to show a sex bias in resistance [7,15,50].

Trends in damselfly body size (measured as wing length) did not explain trends in mite numbers on hosts within a season. Body size was also not correlated with the probability of mounting an immune response in either males or females, a finding also reported in studies with *Lestes forcipatus *[15,44,50]. Wing length increased in both sexes over the study period, but whether this is related to parasitism or is due to other factors is not known.

Although this correlative study suggests that parasite abundance affects resistance in damselflies, other ecological or evolutionary factors could be responsible for the pattern. A limitation of the present study is the lack of experiments to elucidate which factors contribute to the host's ability to resist mites. In addition, although genetic variation for resistance in some animals has been demonstrated [61], it is not known if resistance in damselflies has a genetic component.

## Conclusions

Parasite population size (as indexed by prevalence and intensity measures) related to the expression of host immunity over seven years in this mite-damselfly system. Resistance in the host population increased dramatically and then declined again over a period of a few years. Determining which factors affect variation in immune response has important implications for understanding ecological and evolutionary questions about both host and parasite populations. Our data suggest that factors such as temperature, which have been identified in related laboratory studies as contributing to resistance by similar hosts, do not appear to be important in natural populations over the degree of natural variation that we monitored in this study. The sole factor that contributed to the appearance and expression of resistance in this host population was prevalence and intensity of parasites, suggesting that resistance in these damselflies is a plastic response. This conclusion differs from [62], who conclude that cyclical resistance to a virus in western tent caterpillars is probably genetically based. Although cyclical patterns in parasitism have been documented before [63-65], cycles of resistance in insects are not common (but see [62]), and the factors involved in this complex phenomenon deserve further study.

## Methods

### Study site and relevant natural history of mites and damselflies

Barb's Marsh is a 1-ha, isolated marsh surrounded by hay fields and mixed woods near the Queen's University Biology Station in eastern Ontario, Canada (45°37'N, 76°13'W). The mite *Arrenurus pollictus *is specific to *Lestes disjunctus *at this site. Further, *L. disjunctus *is not parasitized by other mite species there. Larval mites initially challenge the final aquatic larval instars of lestids (where resistance to them takes the form of grooming similar to that in a coenagrionid larvae [66]). They are phoretic on these hosts, but when the damselflies eclose, the mites pierce the host cuticle with their chelicerae and form a blind-ended feeding tube. Mites cannot move to other hosts once feeding begins, so enumerating them upon host emergence provides accurate data on degree of parasitism for individuals.

After a pre-reproductive period of about 12 d (unpublished data), female *L. disjunctus *damselflies lay multiple clutches of eggs at intervals of 1- 5 d [67]. It is during damselfly oviposition and mate guarding that fully engorged mites drop off their hosts (leaving a scar on the damselfly which can become obscured with age). The larval mite then goes through predatory nymphal and adult stages punctuated by quiescent protonymphal and tritonymphal stages.

*L. disjunctus *is the most common lestid damselfly at this site, and dispersal is very low [68]. *Lestes rectangularis *and *L. congener *are present in low numbers and are rarely parasitized by *A. pollictus *(unpublished data.). Female *L. disjunctus *oviposit endophytically; eggs overwinter and hatch in mid-May. Emergence begins in mid-June, and the flight season ends in early August. Resistance occurs within 24 h of host emergence in lestids, and dead mites are always associated with a melanised feeding tube [14,69]. Resistance expression should not be influenced by investment in reproduction, since resistance occurs during the first 24 h to few days of the pre-reproductive period. Surveys of mature damselflies in the weeks after emergence reveal whether or not mites have engorged successfully, or if the damselfly has mounted a melanotic encapsulation response, resulting in dead mites still attached to the host [69].

### Monitoring of damselfly emergence

Ten emergence traps (each 1 m^2^) were placed haphazardly over suitable vegetation at the margins of the marsh (where these damselflies emerge) in the first week of June each year. Teneral damselflies were collected from traps each day, and the number of *A. pollictus *mites attached to each damselfly was recorded using a 20× loupe. The length of the damselfly right forewing (distance between nodus and tip) was measured with digital calipers (± 0.01 mm). Emergence traps were not used in 2004 and 2007. In 2004 however, newly emerged damselflies were caught with hand nets during a portion of the emergence period (June 23-26).

### Surveys of mature damselflies

Adult damselflies were netted daily from late June to early August in a 1-hour survey (1100-1200), except on days when their activity was limited by inclement weather. We recorded host sex and age (young, mature, or old; based on body colouration [70,71]), as well as number of scars, live mites and dead mites. Each damselfly was marked on one of the hindwings with a permanent marker (Stanford^® ^Sharpie) to avoid recounting it if captured in subsequent surveys. In 2004, adults were collected only from July 11-19. In 2007, only two surveys were conducted (July 14,15), and wing length was not recorded.

### Air temperatures

Air temperatures were recorded using a standard Stevenson screen at 1.5 m height with a Campbell Scientific 21× datalogger. Mean daily temperatures taken from hourly averages were assessed prior to the emergence period (May 15 to the first day of emergence) and during the flight season (first day of emergence to August 1).

### Statistical Analyses

Analyses were completed in JMP (version 5.1: SAS Institute, 2002). Logarithmic transformations of wing length and mite intensity were done to satisfy assumptions of normality (actual values are presented in tables and figures). Separate analyses were completed for newly emerged and mature damselflies. For analyses of intensity, mature damselflies with mite scars were excluded because scars can become difficult to see as damselflies age. However, these individuals were included for summaries of all other variables.

Differences in prevalence and resistance between sexes within a year were analyzed with Kruskal Wallis chi-square tests. Multiple comparisons of wing length, prevalence and intensity among years were completed using ANOVA and Tukey Honest Significant Difference (HSD). Because sex was strongly correlated with wing length, we analyzed the sexes separately in a logistic regression model to determine if year, wing length or intensity were significant predictors of resistance. Data from 2007 were excluded because wing length was not measured that year. We used a nominal logistic regression model to determine whether intensity at emergence was a significant predictor of whether resistance was present in the mature damselfly population each year. We also used this model to determine whether intensity in mature damselflies was a significant predictor of whether this sample of the population resisted mites.

## Authors' contributions

LN did fieldwork, data analysis and drafted the manuscript. TR conducted fieldwork and did preliminary data analysis. MRF designed the study, conducted fieldwork each year and revised the manuscript. All authors read and approved the final manuscript.

## Supplementary Material

Additional file 1 **Tables S1-S3**. Tables too large to be uploaded with the main text file (but are required in the main body of the published version). Click here for file
