# Supplementary material for: Inter-annual variation in prevalence and intensity of mite parasitism relates to appearance and expression of damselfly resistance
Source: BMC Ecol. 2010 Feb 14;10:5. doi: 10.1186/1472-6785-10-5 (PMC2829476; doi:10.1186/1472-6785-10-5)
Supplement: Additional file 1 — Tables S1-S3. Tables too large to be uploaded with the main text file (but are required in the main body of the published version). [file 1472-6785-10-5-S1.DOC]

Table S1. Summary of inter-annual variation in numbers (N) of newly emerged *Lestes disjunctus* females (♀) and males (♂), in their mean wing lengths (mm ± s.e.), in start date of the emergence period each year (start date) and in duration of the emergence period in days (d), under the heading “Damselfly hosts”. Summary also of inter-annual variation in prevalence and mean (± s.e.) number of *Arrenurus pollictus* mites per parasitized host (intensity) for newly emerged female and male damselflies (under the heading “Mite parasites”). The maximum number of parasites per host ranged from a low of 20 (in 2008) to a high of 135 (in 2004). Values within a row with different letters indicate significant differences among years using Tukey HSD (for wing length, mean intensity), Clopper-Pearson estimates of confidence levels (for prevalence), and comparisons of cumulative emergence curves (for duration).

2002 2003^1^ 2004^2^ 2005 2006 2008

Damselfly hosts

N 102 393 169 46 44 75

♀ 52 233 95 26 24 41

♂ 36 160 74 18 20 34

$\overline{X}$ wing length

♀ 13.9±0.1^a^ 14.7±0.1^b^ 14.8±0.2^b^ 14.3±0.1^a^ 14.9±0.2^b^ 15.4±0.1^c^

♂ 12.7±0.1^a^ 13.4±0.1^b^ 13.3±0.2 ^b^  13.4±0.1^b^ 13.6±0.1^b^ 14.0 ±0.1^c^

Start date 23 June 21 June - 22 June 12 June 16 June

Duration (d) 9 ^c^ 6 ^b^ - 4 ^a^ 8 ^c^ 9 ^c^

Table 1 cont’d

------------------------------------------------------------------------------------------------------------------------------------------------------------------

Mite parasites

Prevalence 86^a^ 100^b^ 100 96^b^ 100^b^ 77^a^

♀ 89 100 100 96 100 85

♂ 82 100 100 95 100 68

Mean Intensity 10.2±0.9^b^ 9.1±0.4^b^ 54.3±1.8^d^ 17.6±2.4^b^ 26.1±2.8^c^ 5.3±0.5^a^

♀ 10.8±1.4 10.4±0.6 59.3±2.6 22.2±4.1 31.6±4.2 5.5±0.7

♂ 9.2±1.5 7.3±0.5 47.8±2.1 10.9±2.4 19.4±3.1 5.2±0.9

^1^significant female biased sex ratio at emergence in this year

^2^see text for sampling limitations in this year

Table S2. Summary of inter-annual variation in numbers (N) of sexually mature *Lestes disjunctus* females (♀) and males (♂), under the heading “Damselfly hosts”. Also included is the percentage (%) of hosts with one or more dead mites (resistance) and the mean hourly temperature ($\overline{X}$ temp) during the flight seasons for each year. Values within a row with different letters indicate significant differences among years using Tukey HSD (for $\overline{X}$ temp).

2002 2003 2004^2^ 2005 2006 2007^2^ 2008

Damselfly hosts

N 283 355 133 180 130 52 332

♀ 97 100 53 118 73 21 120

♂ 186 255 80 62 57 31 212

Resistance^1^ 0 0 12.8 6.7 3.8 9.6 3.9

$\overline{X}$ temp (°C) 22.6^b^ 21.6^b^ 19.7^a^ 23.1^c^ 21.8^b^  22.1^b^ 19.8^a^

^1^does not include individuals with scars (or without any mites); calculated as % of damselflies in survey sample with one or more dead mites

^2^see text for sampling limitations in these years

Table S3. Overall numbers of mature hosts sampled (N) and the numbers of those showing resistance (NR) in a given year. Both N and NR include hosts with scars (evidence of past parasitism). The NR data are further broken down into mean intensity of parasitism for non resistant versus resistant hosts (females and males combined) and mean wing length (± 1 s.e.) for non resistant versus resistant hosts (females and males considered separately). Non parasitized individuals were omitted from calculations, which results in sample sizes lower than those reported in Table S2. Parasitized damselflies with scars were excluded from the comparison of mean intensity, which results in lower sample sizes than those reported in N.

Year N NR Mean Intensity^3^ (*n*) Wing length (mm)

Non resistant Resistant Non resistant Resistant

2002 222 0 13.4±0.9(141) -

female 14.1±0.1 -

male 12.7±0.1 -

2003 317 0 13.2±0.9(205) -

female 14.7±0.1 -

male 13.5±0.1 -

2004 131 17 32.4±2.2(101) 42.7±9.6(6)

female 6 14.9±0.1 15.3±0.3

male 11 13.5±0.1 13.6±0.2

2005 149 12 17.3±1.7(103) 20.8±6.4(8)

female 8 14.5±0.1 14.4±0.1

male 4 13.1±0.1 13.3±0.4

2006 121 5 23.5±2.2(94) 17.5±3.5(2)

female 3 14.7±0.1 15.3±0.1

male 2 13.2±0.1 13.5±0.3

2007 42 5 8.2±1.0(24) 14.8±4.2(4)

female 3 - -

male 2

2008 234 13 4.4±0.4(130) 4.7±1.8(6)

female 2 14.2±0.1 13.9±0.3

male 11 13.5±0.2 13.4±0.3
